# Impairment of social behaviors in *Arhgef10* knockout mice

**DOI:** 10.1186/s13229-018-0197-5

**Published:** 2018-02-13

**Authors:** Dai-Hua Lu, Hsiao-Mei Liao, Chia-Hsiang Chen, Huang-Ju Tu, Houng-Chi Liou, Susan Shur-Fen Gau, Wen-Mei Fu

**Affiliations:** 1 Pharmacological Institute, College of Medicine, National Taiwan University, Taipei, Taiwan; 2 Department of Psychiatry, National Taiwan University Hospital and College of Medicine, Taipei, Taiwan; 3 Department of Psychiatry, Chang Gung Memorial Hospital Linkou, Taoyuan, Taiwan; 4 Department and Graduate Institute of Biomedical Sciences, Chang Gung University, Taoyuan, Taiwan

**Keywords:** ARHGEF10, Autism spectrum disorder, Social deficits, Serotonin, Norepinephrine

## Abstract

**Background:**

Impaired social interaction is one of the essential features of autism spectrum disorder (ASD). Our previous copy number variation (CNV) study discovered a novel deleted region associated with ASD. One of the genes included in the deleted region is *ARHGEF10*. A missense mutation of *ARHGEF10* has been reported to be one of the contributing factors in several diseases of the central nervous system. However, the relationship between the loss of ARHGEF10 and the clinical symptoms of ASD is unclear.

**Methods:**

We generated *Arhgef10* knockout mice as a model of ASD and characterized the social behavior and the biochemical changes in the brains of the knockout mice.

**Results:**

Compared with their wild-type littermates, the *Arhgef10*-depleted mice showed social interaction impairment, hyperactivity, and decreased depression-like and anxiety-like behavior. Behavioral measures of learning in the Morris water maze were not affected by *Arhgef10* deficiency. Moreover, neurotransmitters including serotonin, norepinephrine, and dopamine were significantly increased in different brain regions of the *Arhgef10* knockout mice. In addition, monoamine oxidase A (MAO-A) decreased in several brain regions.

**Conclusions:**

These results suggest that *ARHGEF10* is a candidate risk gene for ASD and that the *Arhgef10* knockout model could be a tool for studying the mechanisms of neurotransmission in ASD.

**Trial registration:**

Animal studies were approved by the Institutional Animal Care and Use Committee of National Taiwan University (IACUC 20150023). Registered 1 August 2015.

**Electronic supplementary material:**

The online version of this article (10.1186/s13229-018-0197-5) contains supplementary material, which is available to authorized users.

## Background

Autism spectrum disorder (ASD) is a common neurodevelopmental disorder marked by lifetime social functional impairment [1, 2]. The essential features of ASD include impairment in reciprocal social communication; a deficit in communication ability; and restricted, repetitive behavior and interests [1]. ASD occurs at a higher incidence in males than females, with a common consensus ratio of 4:1 [3]. The prevalence of ASD has been estimated at approximately 1.5% and has increased dramatically over the past few decades [4, 5]. Various genetic studies have provided convincing evidence that ASD is a complex and highly polygenic disease [6]. However, the genetic underpinnings of ASD remain unclear, which impedes understanding of the disease pathology and the search for treatments.

Our previous study identified two novel chromosomal deletions in two unrelated ASD patients [7]. One of the deletions, which spans 8p23.3-pter, contains three genes––*DLGAP2*, *CLN8*, and *ARHGEF10*––that may be relevant to neurological functions. The functional loss of these genes might contribute to the clinical symptoms of ASD [8, 9]. In this study, we target the gene *ARHGEF10* to further investigate the impacts of its functional loss. ARHGEF10, as a rho guanine nucleotide exchange factor (GEF), regulates rho GTPases by catalyzing the exchange of G-protein-bound GDP for GTP. There are over 60 rho GEFs identified in the human genome. However, few have been functionally evaluated in animal models. The Thr109lle mutation of the rho GEF 10 (*ARHGEF10*) gene was found in a family of patients with slow nerve conduction and thinly myelinated peripheral nerves [10]. In addition to its possible role in the myelination process, the single nucleotide polymorphism of ARHGEF10 has also been reported to be associated with schizophrenia [11]. GEFs are the main regulators that facilitate the activation of rho GTPases by converting them from the GDP-bound state to an active GTP-bound state. Rho GTPases have been widely studied in neuronal development and neuronal diseases [12]. Given the importance of rho GTPases in the nervous system, dysregulation of rho GEFs is believed to be involved in neurodevelopmental diseases. For example, a mutation of *ARHGEF6* has been associated with intellectual disability [13]. These findings encouraged us to further study the possible role of *ARHGEF10* in ASD.

To explore the role of *ARHGEF10* in ASD and to further understand the impacts of *ARHGEF10* deletion on the molecular mechanisms of neurological function, we generated an *Arhgef10* knockout mouse model by deleting exons 4 and 5 of the mouse *Arhgef10* gene. The mice without the *Arhgef10* gene have normal fertility and body weight gain. The *Arhgef10* knockout mice were then subjected to tests measuring their startle responses, motor behavior, spatial learning, and social behavior. Behavioral changes may reflect a disturbance of neurotransmission. Previous studies have shown that the dysregulation of biosynthesis, transportation, and degradation of neurotransmitters could be associated with ASD [14]. In addition, genetic association studies have identified some genes that encode the transporters or degradation enzymes of neurotransmitters as contributing to the risk of ASD [15]. For instance, monoamine oxidase A (MAO-A), an enzyme that is important for the metabolism of serotonin and norepinephrine, is associated with ASD in a population-based study [15]. Another population-based association study also indicates that changes in monoamine oxidase B (MAO-B) activity increase ASD risk in males [16]. Based on those findings, we evaluated the levels of monoamines in *Arhgef10* knockout mice to understand the possible roles of *Arhgef10* in the serotonergic system.

In this study, we found that *Arhgef10* knockout mice exhibited impaired social interaction and social recognition, representing the key characteristics of ASD. In addition, *Arhgef10* knockout mice also displayed reduced anxiety-like and depression-like behaviors and increased locomotor activity. Additionally, serotonin, norepinephrine, and dopamine were elevated in the frontal cortex, hippocampus, and amygdala in *Arhgef10* knockout mice. Moreover, MAO-A, a molecule that regulates the levels of certain neurotransmitters, was also reduced in *Arhgef10* knockout mice. Behavioral studies and biochemical examination showed that ARHGEF10 not only plays a critical role in social behavior but also participates in the regulation of neurotransmitters.

## Methods

### Animals and experimental design

*Arhgef10* knockout mice were generated by the deletion of exon 4 and exon 5 using the Cre-loxP site-specific knockout according to a method described previously [8]. The strategy for generating *Arhgef10* knockout mice is shown in Additional file 1: Figure S1. In brief, exons 4 and 5 of ARHGEF10 in embryonic stem cells from the 129S1/Sv mouse strain were replaced with a construct containing ARHGEF10 exons 4 and 5 interposed between two loxP sites and a NEO cassette to produce Cre-induced homologous recombination. *Arhgef10* knockout mice were then backcrossed for at least ten generations with C57BL/6J mice. The *Arhgef10* knockout mice used in this study were produced by heterozygous breeding pairs in a trio breeding format. All mice were kept under standard temperature, humidity, and timed lighting conditions and provided with mouse chow and water ad libitum. *Arhgef10* knockout mice and their control littermates were housed in groups (3–5 mice per cage).

Behavioral tests were conducted during the light cycle (07:00–19:00) in a testing room next to the mouse housing room. Eight- to twelve-week-old male *Arhgef10* KO mice and their WT littermates were used in this study. All behavioral tests were carried out with male mice. Animals in the same littermates were used in the same behavioral test, except for the plus maze, open field, and water maze tests. In these three tests, the intervals between tests were approximately 7 days each. The open field test was conducted first, followed by the plus maze and then the water maze. Mice were transported to the testing room and habituated for 30 min before behavioral testing. All animal experiments were approved by the Ethical Committee for the Animal Research of National Taiwan University.

### Sample preparation

Mice were anesthetized with isoflurane and then decapitated. For Nissl staining, the brains of 10-week-old mice were removed after saline perfusion and post-fixed with 4% paraformaldehyde (PFA) overnight. Tissue samples for Western blots and high-performance liquid chromatography (HPLC) were prepared by dissecting four parts from the fresh brains of 10- to 12-week-old mice: frontal cortex, striatum, hippocampus, and amygdala. These tissues were then weighed and homogenized.

### Nissl staining

Nissl staining was performed as previously described [17]. In brief, 40-μm-thick frozen brain sections from WT and *Arhgef10* knockout mice were mounted on gelatin-coated slides and air-dried overnight. The slices were then placed directly into xylene for 3 min and then rehydrated with 100, 95, and 85% alcohol, followed by distilled water. The slices were stained with 0.02% crystal violet (Sigma-Aldrich, St. Louis, MO, USA) solution for 25–35 min and then rinsed quickly in distilled water. Finally, the slices were soaked in xylene and mounted with permanent mounting medium.

### Open field test

Mice were placed in an open plastic chamber (40 × 40 × 38 cm), and locomotor activity was monitored for 1 h. The central region of the open field was defined as a region of 20 × 20 cm^2^, and all activity was measured by a 16 × 16 photobeam sensor connected to an automated tracking program (PAS-Open Field, San Diego Instruments). The number of beam breaks were used as an indicator of locomotor activity.

### Elevated plus maze test

The elevated plus maze (EPM) apparatus (made of white Plexiglas) was elevated 40 cm above the floor and consisted of two open arms alternating with two closed arms. The open arms and closed arms were made of plastic and measured 25 cm long × 5 cm wide. The two closed arms were protected by 15-cm-high walls. Mice were placed in the EPM for 5 min, and the time spent in each arm and frequency of entry into each arm were recorded by Noldus EthoVision 3.0 (EthoVision^®^, Noldus Information Technology).

### Tests for sociability and social novelty preference in a three-chamber apparatus

The tests for sociability and social novelty preference were performed in a three-chamber apparatus as previously described with minor modification [18, 19]. The animals used here were all age- and sex-matched littermates; C57BL/6J mice were used as the stranger mice. All animals were habituated to the test chamber for 30 min 1 day before the behavioral test. The three-chamber apparatus was a 21 cm (height) × 25 cm (width) × 48 cm (length) black plastic box. Before the sociability test, the animal was free to explore the apparatus for 5 min. In the sociability test session, an unfamiliar same-sex mouse, designated as stranger 1, was placed in the plastic cylinder; an empty cylinder was placed in the opposite chamber. The cylinders (13 cm in height, 10 cm in diameter) were transparent, and their walls contained holes that allowed the mice to sniff each other. The test mouse was placed in the central chamber and allowed to freely explore these three chambers for 10 min. In the social novelty preference test, a second novel mouse, stranger 2, was placed in the opposite chamber, which was empty in the previous session. The test mouse was allowed to freely explore the chambers for 10 min in this session. The time spent in each chamber and the time spent sniffing or interacting with the stranger mouse were recorded. In addition, the number of entries into all chambers were analyzed using EthoVision.

### Tail suspension test and forced swim test

The tail suspension test (TST) [20] and the forced swim test (FST) are the most commonly used tests to evaluate depression-like behavior in rodents. In the TST, the animals were suspended by the tail with adhesive tape for 6 min. In the FST, the animals were placed in a plastic cylinder tank (30 cm in height × 15 cm in diameter) filled with 25 ± 2 °C water for 6 min. The complete TST and FST sessions were all videotaped for analysis. Immobility was defined as a lack of motion of the whole body at 2–6 min, when the mice ceased struggling and began passively floating, making only the movements necessary to keep the head above water.

### Pre-pulse inhibition test

Pre-pulse inhibition (PPI) was tested in an acoustic startle chamber (SR Lab, San Diego Instruments, San Diego, CA). The chamber contained a Plexiglas cylinder (12.6 cm in length × 4.0 cm in internal diameter) fixed on a platform under which a piezoelectric accelerometer recorded and transduced the motion of the tube. The sensor transmitted the digitized signals to a computer interfaced with the startle apparatus. To provide a consistent acoustic environment and mask external noise, we maintained a continuous background of 65-dB white noise within each chamber throughout the PPI tests. The inter-trial interval was between 10 and 20 s. Each startle trial consisted of a single 120-dB white noise burst lasting 40 ms. Each PPI trial consisted of a pre-pulse (20 ms burst of white noise with an intensity of 70, 74, or 82 dB) followed by the startle stimulus 100 ms later (120 dB, 40 ms of white noise). Each of the three types of pre-pulse trials (70, 74, and 82 dB) was presented 10 times. The percentage of PPI was calculated according to the following formula: %PPI = (S − (P + S))/S × 100%, where P + S is the recorded response amplitude for pre-pulse plus startle pulse trials, and S is the recorded response amplitude for startle pulse-only trials.

### Morris water maze test

Spatial learning was evaluated behaviorally using the Morris water maze. The maze consisted of a circular pool of water, 105 cm in diameter and 21 cm in height. The pool was divided into four equal quadrants: the target zone, two adjacent zones, and the opposite zone. The pool was filled with water at 22 ± 0.5 °C. A circular platform of 10 cm diameter was placed at the center of the target quadrant, and approximately 1.5 cm below the surface of the water. The maze was surrounded by four simple visual cues external to the maze. The movements of the mice were recorded with a video camera placed on the ceiling over the center of the maze, and the paths of the mice were analyzed using EthoVision. The training was started by acclimating the mice to the task environment with 1 day of free swimming in the pool without the platform. Each mouse underwent four consecutive training trials per day for 4 days. In each trial, the mice were placed along the edge of one quadrant of the maze; all quadrants except the target quadrant were used as starting locations. The duration from the time when the mouse entered the water until it climbed onto the platform was recorded as escape latency, and the mean latency to find the hidden platform was calculated for each individual mouse on each day. The maximum duration of each trial was 1 min, and the mice were removed from the pool by the experimenter after each trial. If the mice failed to find the platform within 1 min, they would be placed on the platform, where they were allowed to remain for 15 s. For the probe trial, 24 h after the final trial, the platform was removed. Each mouse was placed in the water maze at the edge of the former platform location and allowed to swim freely for 60 s. The total time that each mouse spent in each quadrant of the tank was recorded.

### High-performance liquid chromatography

The levels of serotonin, norepinephrine, and dopamine in the frontal cortex, striatum, hippocampus, and amygdala were measured by HPLC analysis. Four different mouse brain areas were dissected from the cerebral hemisphere, weighed, and homogenized in 0.1 N perchloric acid by sonication. The homogenates were then centrifuged at 14,500×*g* for 30 min at 4 °C, and the supernatants were collected for HPLC analysis. HPLC–ECD (Sykam, Gilching, Germany) was used to evaluate levels of monoamines, including dopamine, 5-HT, and NE and their metabolites, in the samples (20 μl). The chromatogram peaks corresponding to the monoamines were identified by their retention times compared with the elution times of monoamine standards (Sigma, St. Louis, MO). The levels of dopamine, 5-HT and NE, were then estimated by the ratios of the peak heights to those of the internal standards and expressed as nanograms of neurotransmitter per gram of tissue.

### Western blotting

Western blot analysis was performed to characterize ARHGEF10 (Proteintech Group, IL, USA; 11112-1-AP), GAPDH (Santa Cruz, Dallas, TX, USA; sc25778), β-actin (Sigma-Aldrich, St. Louis, MO, USA; A5316), MAO-A (Abcam, Cambridge, UK; ab126751), monoamine oxidase B (MAO-B) (Abcam, Cambridgeshire, UK; ab137778), dopamine ß-hydroxylase (DβH) (Abcam, Cambridge, UK; ab108384), and tryptophan hydroxylase (TPH) (Abcam, Cambridge, UK; ab52954) expression in the brains of WT and ARHGEF10 knockout mice. Mouse brain tissues were lysed in RIPA buffer (150 mM NaCl, 50 mM Tris–HCl, 1 mM EGTA, 1% Nonidet P-40, 0.25% deoxycholate, 1 mM sodium fluoride, 50 mM sodium orthovanadate) supplemented with Halt protease inhibitor cocktail (Thermo, IL, USA). Proteins were separated by SDS-PAGE, transferred to PVDF membranes, and blocked with 5% non-fat milk for 1 h. The membranes were then incubated with primary antibodies at 4 °C overnight followed by the appropriate secondary antibodies at room temperature for 1 h. The protein bands were visualized using enhanced chemiluminescence (ECL) (Millipore, MA, USA) reagent, and blot images were captured using a UVP imaging system with LabWorks Software (Upland, CA, USA).

### Data analysis

Throughout the study, parametric analysis was performed by one- or two-way ANOVA, using a between-group factor of genotype and a within-group factor of each behavioral parameter. If the overall ANOVA showed a significant difference, Tukey’s test was used for post hoc comparisons. Statistical analysis was conducted using SPSS (IBM Inc., Somers, NY, USA) and GraphPad Prism (San Diego, CA, USA) software. Graphs of the data were also created using GraphPad Prism. The data were represented as the mean ± standard error.

## Results

### ARHGEF10 expression in different brain regions

**Fig. 1:**
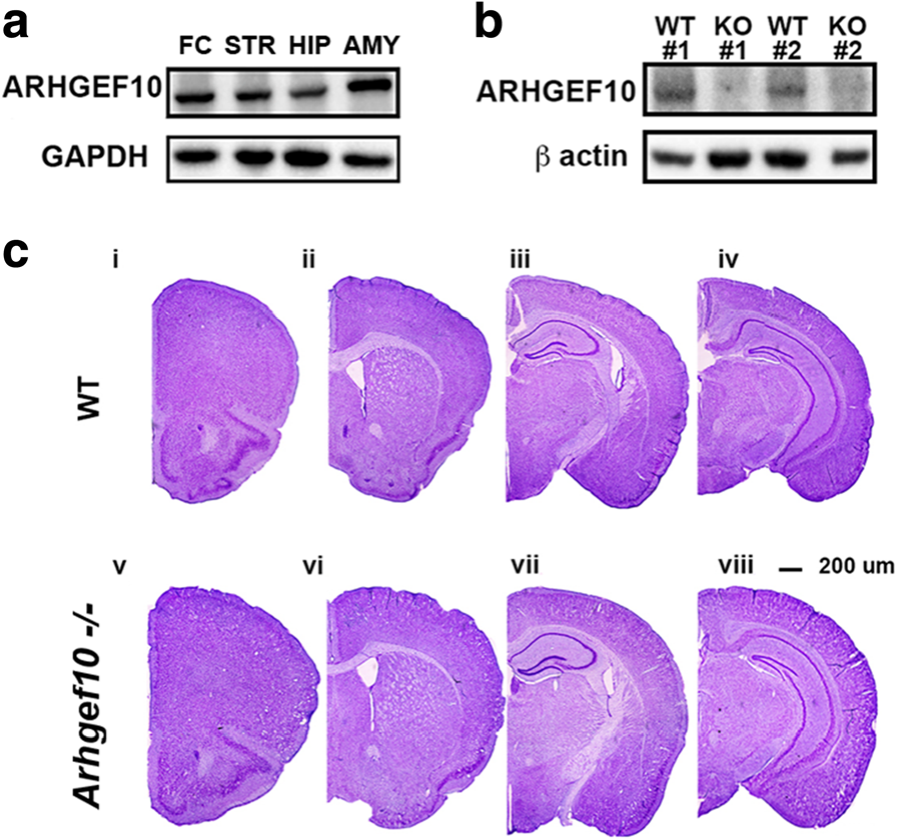
ARHGEF10 protein expression, and Nissl staining of the adult brain in WT and *Arhgef10* knockout mice ARHGEF10 protein expression, was measured by Western blotting. **a** ARHGEF10 protein was widely expressed in the frontal cortex, striatum, hippocampus, and amygdala. **b** Western blot of ARHGEF10 from the whole brains of WT and *Arhgef10* knockout mice. **c** Nissl staining of brain sections from 2-month-old (adult) WT (i–iv) and *Arhgef10* ^−^/^−^ mice (v–viii). *FC* frontal cortex, *STR* striatum, *HIP* hippocampus, *AMY* amygdala, *WT* wild-type, *KO Arhgef10* knockout

To examine ARHGEF10 expression in the central nervous system, we measured the protein level by Western blot analysis. As shown in Fig. 1a, ARHGEF10 protein was widely expressed in the wild-type (WT) mouse brain, especially in the frontal cortex and amygdala. Notably, the absence of ARHGEF10 protein in the knockout mouse brain was confirmed by Western blotting (Fig. 1b). Nissl staining of serial coronal brain sections revealed a similar brain structure between WT and *Arhgef10* knockout mice (Fig. 1c).

### *Arhgef10* knockout mice display social deficits in the three-chamber test

**Fig. 2:**
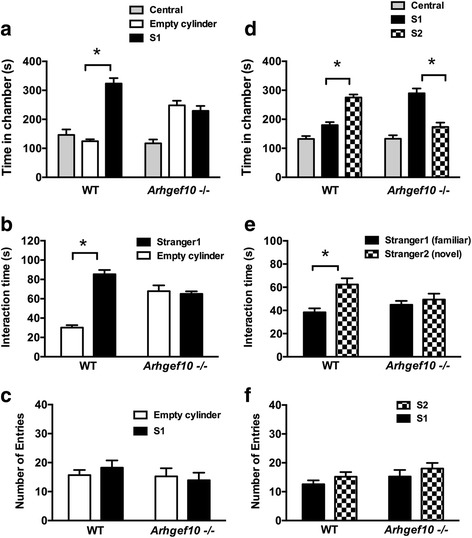
*Arhgef10* knockout mice exhibit social impairment in the three-chamber test. **a** The time spent in each of the three compartments was analyzed using EthoVision. **b** The active interaction times with the empty cylinder and an unfamiliar mouse (stranger 1) in the session were also evaluated. WT mice (*n* = 12) spent much more time in the chamber with stranger 1 than in the empty cylinder, and displayed more interaction with the mouse than with the empty cylinder, indicating normal sociability (*p* < 0.001). However, *Arhgef10* ^−^/^−^ mice (*n* = 12) spent equal durations of both total time and active interaction time in the chamber with empty cylinder and stranger 1. **d** In the preference for social novelty test, the time spent in each chamber was analyzed as in the previous test. WT mice spent more time in the chamber with a novel mouse (stranger 2) than in the compartment with stranger 1. However, Arhgef10 −/− mice spent less time in the chamber with stranger 2 than in the chamber with stranger 1. **e** The duration of contact time was measured with stranger 1 and the novel mouse stranger 2. WT mice displayed a significant increase in the duration of close interaction with stranger 2 compared with stranger 1 (*p* < 0.001). However, Arhgef10 −/− mice did not show a preference for novel stranger 2

To explore whether *Arhgef10* knockout affects social interaction, we employed the three-chamber paradigm to test sociability and social recognition. After habituation, a novel same-sex mouse was placed within the plastic cylinder (stranger mouse 1) in one chamber, and one empty cylinder was placed in another chamber. We found that WT mice spent a significantly longer time in the chamber with a stranger mouse than in the empty chamber (Fig. 2a; *F*_1,22_ = 36.517, *p* < 0.001 for genotype × chamber by two-way ANOVA; *F*_1,22_ = 24.831, *p* < 0.001 for chamber; Tukey’s post hoc comparisons were used to examine the differences between the empty cylinder and stranger mouse 1, WT: *p* < 0.05; KO: *p* > 0.05; *n* = 12 for each). However, *Arhgef10* knockout mice did not spend a longer time in the chamber with the stranger mouse (Fig. 2a). Upon further evaluation of the social interactions, we found that WT mice spent significantly and markedly more time in close interaction with the stranger mouse by sniffing the holes of the cylinder, indicating normal social ability (Fig. 2b; *F*_1,22_ = 52.971, *p* < 0.001 for genotype × chamber by two-way ANOVA; *p* < 0.05 for post hoc comparisons between the empty cylinder and the stranger mouse, *n* = 12). *Arhgef10* knockout mice showed no significant preference between these two cylinders, indicating that they did not exhibit interest in the stranger mouse (Fig. 2b; *p* > 0.05 for post hoc comparisons between the empty cylinder and the stranger mouse, *n* = 12). Since enhanced locomotor activity may increase the possibility of contacts between mice, we further examined the number of entries into each chamber. We found that the number of entries into these two chambers were similar in WT mice and *Arhgef10* knockout mice (Fig. 2c; *F*_1,22_ = 1.607, *p* = 0.218 for genotype × chamber, by two-way ANOVA).

In the test for social novelty preference, we further examined social recognition in WT and *Arhgef10* knockout mice. Mice naturally exhibit a preference for social novelty, spending more time with a new mouse than with a familiar mouse as a social stimulus. In addition to stranger 1 in the original cylinder, another stranger mouse (stranger 2) was placed in the second cylinder. WT mice showed a preference for exploring the compartment with the novel mouse, stranger 2, compared with the chamber containing stranger 1 (Fig. 2d and e; *F*_1,22_ = 5.843, *p* = 0.024 for genotype × chamber, by two-way ANOVA; post hoc for comparisons between stranger 1 and stranger 2 revealed *p* < 0.05, *n* = 12 for WT, and *p* < 0.05, *n* = 12 for KO). Although *Arhgef10* knockout mice spent more time in the chamber with the first stranger mouse than in the chamber with the second stranger mouse (Fig. 2d), the time spent in social interactions, such as sniffing or tail rattling or time spent near the cylinder containing the stranger mice was similar between the first and the second stranger mouse (Fig. 2e). Keeping up with the decreased social interaction in sociability test, it was found that the social novelty preference was affected in *Arhgef10* knockout mice. However, the longer time spent in the chamber with the first stranger mouse without more social interaction may imply that *Arhgef10* knockout mice need more time to become familiar with the stranger mouse during the experiment. Both WT and *Arhgef10* knockout mice showed a comparable number of entries into the compartment with stranger 1 and the one with the novel mouse, stranger 2 (Fig. 2f; *F*_1,22_ = 0.983, *p* = 0.602 for genotype × chamber, by two-way ANOVA).

### Increased locomotor activity in *Arhgef10* knockout mice

**Fig. 3:**
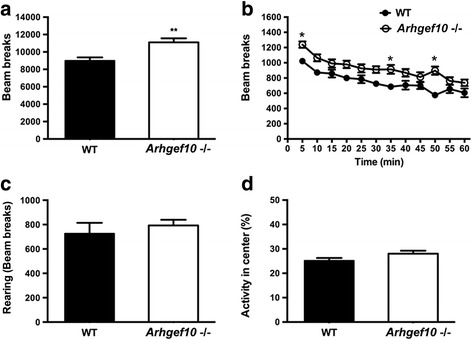
*Arhgef10* knockout mice display spontaneous locomotor hyperactivity in the open field test. Spontaneous locomotor activity was tested in an open field for 60 min. **a** Total ambulatory locomotion (*p* = 0.001). **b** Time course of locomotor activity. Note that locomotor activity was greater in *Arhgef10* knockout mice. **c** No difference between WT mice and *Arhgef10* ^−^/^−^ mice in total rearing was observed. **d** No difference in percent of activity in the central part of the open field was observed between WT mice and *Arhgef10* ^−^/^−^ mice. The bar graph shows mean ± SEM (*n* = 16 per genotype for WT mice and KO mice); * *p* < 0.05 compared with WT mice by one-way ANOVA

The open field test was used to evaluate the general locomotor and exploratory activity of the mice. *Arhgef10* knockout mice showed a higher level of locomotor activity in open field (*F*_1, 30_ = 12.296, *p* = 0.001, *n* = 16) (Fig. 3a). In a 60-min open field test, *Arhgef10* knockout mice exhibited significantly enhanced locomotor activity compared with WT mice at 0–5, 30–35, and 45–50 min (Fig. 3b, Tukey’s test for multiple comparisons, *p* < 0.05). There was no significant difference in rearing activity between WT and knockout mice (Fig. 3c). In addition, there was no significant difference between WT and *Arhgef10* knockout mice in the amount of activity occurring in the center of the field (Fig. 3d).

### Reduction of anxiety-like behavior in *Arhgef10* knockout mice

**Fig. 4:**
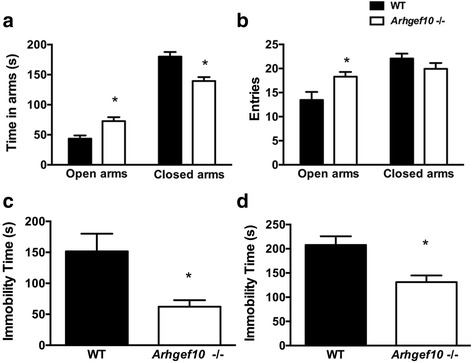
Reduced anxiety-like and depression-related behavior in *Arhgef10* ^−^/^−^ mice. Anxiety-like behavior was tested in an elevated plus maze (EPM) for 5 min. For depression-related behavior, immobility was measured between 2 and 6 min in the forced swim test (FST) and the tail suspension test (TST). **a** *Arhgef10* ^−^/^−^ mice spent more time in open arms than WT mice (*n* = 13 for each) in the EPM. **b** Total entries into open arms and closed arms of the EPM in WT and *Arhgef10* ^−^/^−^ mice. **c** Reduced immobility time was observed in *Arhgef10* ^−^/^−^ mice (*n* = 9 and 10 for WT and KO, respectively) during the FST. **d** *Arhgef10* ^−^/^−^ mice also displayed reduced immobility time compared with WT mice during the TST (*n* = 9 and 10 for WT and KO, respectively). The bar graph shows the mean ± SEM. *p < 0.05 compared with WT

The EPM was used to measure the level of anxiety-like behavior in the mice. The time spent in the open arms or closed arms is used as an index to define the level of anxiety-like behavior. It was found that *Arhgef10* knockout mice displayed less anxiety-like behavior. The time spent in the open arms was significantly increased in *Arhgef10* knockout mice (open arms: *F*_1,23_ = 11.04, *p* = 0.003; closed arms: *F*_1,23_ = 14.481, *p* = 0.001, *n* = 13 for WT vs *Arhgef10* KO) (Fig. 4a), indicating that anxiety-like behavior was reduced in knockout mice. Upon analyzing the number of entries into each arm, we found that the number of entries into the open arms was significantly increased in *Arhgef10* knockout mice (open arms: *F*_1,23_ = 6.301, *p* = 0.02; closed arms: *F*_1,23_ = 2.431, *p* = 0.134 for WT vs *Arhgef10* KO) (Fig. 4b). The results demonstrated that there was a reduction of anxiety-like behavior in *Arhgef10* KO mice.

### Reduction of depression-like behavior in *Arhgef10* knockout mice

To further examine whether other mood-related behaviors were also affected in *Arhgef10* knockout mice, animals were subjected to the tail suspension test (TST) and the forced swim test (FST). Increased immobility and floating time are indicative of depression-related behavior. Interestingly, *Arhgef10* knockout mice showed a significant reduction of immobility in both the FST (151.1 ± 9.47 s, and 62.43 ± 1.5 s for WT and KO mice, respectively) and TST (207.0 ± 6.013 s and 131.2 ± 13.47 for WT and KO, respectively) (Fig. 4c, d). The duration of immobility time in both the FST and the TST was significantly shorter in *Arhgef10* knockout mice than in WT mice (*F*_1,17_ = 39.175, *p* < 0.0001 in FST; *F*_1,17_ = 24.109, *p* = 0.0013 in TST; *n* = 9 and 10 for WT and KO, respectively). These results indicated that there was reduction of depression-like behavior in *Arhgef10* KO mice.

### Pre-pulse inhibition is unaffected in *Arhgef10* knockout mice

**Fig. 5:**
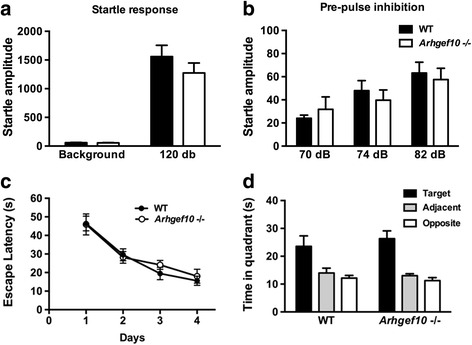
*Arhgef10* knockout does not affect the startle response, pre-pulse inhibition (PPI) or learning. The startle reflex response was measured as startle amplitude, and PPI is the reduction of this response when an acoustic startle (120 dB) is preceded by a stimulus of 70, 74, or 82 dB. **a** There was no difference between WT and *Arhge*^*f1*^*0* ^−^/^−^ mice in startle responses to a 120-dB acoustic stimulus. **b** *Arhgef10* ^−^/^−^ mice did not show a PPI deficit for a pre-pulse of 70, 74, or 82 dB (*n* = 5–6). **c** In the Morris water maze test, all mice were trained for 4 days to reach the platform. **d** On day 5, the platform was removed for the probe test to examine the memory of the animals. Note that *Arhgef10* ^−^/^−^ mice exhibit the same normal acquisition curve as WT mice. Both WT and *Arhgef10* ^−^/^−^ mice spent much more time in the target zone than in any other zone in the probe test. The bar graph shows the mean ± SEM (*n* = 6–7). *p < 0.05 compared with adjacent or opposite quadrants

The pre-pulse inhibition (PPI) test is used to evaluate sensory gating in mice. Acoustic startle responses provide information on the sensorimotor processes of the animal in response to acoustic stimuli. *Arhgef10* knockout mice had a normal startle amplitude in response to 120 dB acoustic stimuli, indicating that the reflexive contraction of the muscles in response to acoustic stimuli was normal (*F*_1.9_ = 1. 171, *p* = 0.337, *n* = 5–6) (Fig. 5a). Moreover, *Arhgef10* knockout mice also exhibited a reduced acoustic startle response when the acoustic startle stimulus was preceded by a weaker acoustic stimulus, indicating a normal PPI response in comparison with WT mice (two-way ANOVA for genotype × pre-pulse dB, main effect of genotype: *F*_1.9_ = 1. 0366, *p* = 0.8524; main effect of pre-pulse dB: *F*_2,18_ = 25.27, *p* < 0.001.) (Fig. 5b).

### *Arhgef10* knockout mice exhibit normal spatial learning in the Morris water maze test

Spatial learning behavior was measured using the Morris water maze test. For four training days, *Arhgef10* knockout mice displayed normal learning ability with typical decreases in escape latency (*F* = 0.260, *p* = 0.618, *n* = 6–7) (Fig. 5c). In the probe test, both WT and *Arhgef10* knockout mice spent much more time in the target quadrant than in opposite or adjacent quadrants (Fig. 5d), indicating that *Arhgef10* knockout mice exhibited normal spatial learning.

### Increased norepinephrine (NE) and serotonin (5-HT) levels in the frontal cortex and amygdala of *Arhgef10* knockout mice

**Fig. 6:**
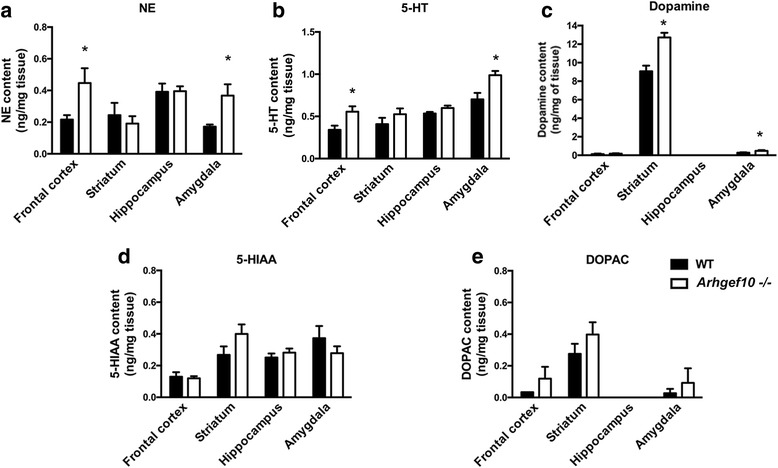
Increased amine levels in the brains of *Arhgef10* knockout mice. The frontal cortex, striatum, hippocampus, and amygdala were dissected from mice brains for HPLC analysis. **a** The content of NE was elevated in both the frontal cortex and the amygdala in *Arhgef10* knockout mice. **b** The content of 5-HT in the frontal cortex and amygdala was significantly increased in *Arhgef10* knockout mice. **c** The content of dopamine was increased in the striatum of *Arhgef10* knockout mice. **d**, **e** There was no significant difference in the content of 5-HIAA or DOPAC (metabolites) between WT and *Arhgef10* −/− mice. The bar graph shows the mean ± SEM (*n* = 6 for each); *p < 0.05 compared with WT

To further explore the possible underlying mechanisms leading to the behavioral changes caused by functional loss of ARHGEF10, we investigated the neurochemical composition of different brain regions of *Arhgef10* knockout mice. The frontal cortex, striatum, hippocampus, and amygdala from WT and KO mice were analyzed by HPLC with electrochemical detection (ECD) to determine the content of dopamine, 5-HT, NE, and their metabolites. The content of NE in the frontal cortex and amygdala was significantly elevated in *Arhgef10* knockout mice (two-way ANOVA for genotype × brain regions, main effect of genotype: *F*_1, 10_ = 6.776, *p* = 0.0264 and brain regions *F*_3, 30_ = 3.250, *p* = 0.0354; interaction: *F*_3, 30_ = 2.764, *p* = 0.0591). Post hoc comparisons between WT and KO revealed significant differences in the frontal cortex and amygdala (Fig. 6a). Serotonin content in the amygdala and hippocampus was also increased in *Arhgef10* knockout mice compared with WT mice (two-way ANOVA for genotype × brain regions, main effect of genotype: *F*_1, 10_ = 11.57, *p* = 0.0068 and brain regions (*F*_3, 30_ = 24.650, *p* < 0.0001; interaction: *F*_3, 30_ = 1.814, *p* = 0.1659). Post hoc comparisons between WT and KO revealed significant differences in the frontal cortex and amygdala. Dopamine in the striatum was also increased in *Arhgef10* knockout mice compared with WT mice (two-way ANOVA for genotype × brain regions, main effect of genotype: *F*_1, 10_ = 23.47, *p* = 0.0007 and brain regions *F* (3, 30) = 731.5, *p* < 0.0001; interaction: *F*_3, 30_ = 20.36, *p* < 0.0001). Post hoc comparisons between WT and KO revealed a significant difference in the striatum (Fig. 6c). However, there were no differences in the metabolites of these monoamines between WT and *Arhgef10* knockout mice (Fig. 6d, e).

### Reduction of MAO-A expression in *Arhgef10* knockout mice

**Fig. 7:**
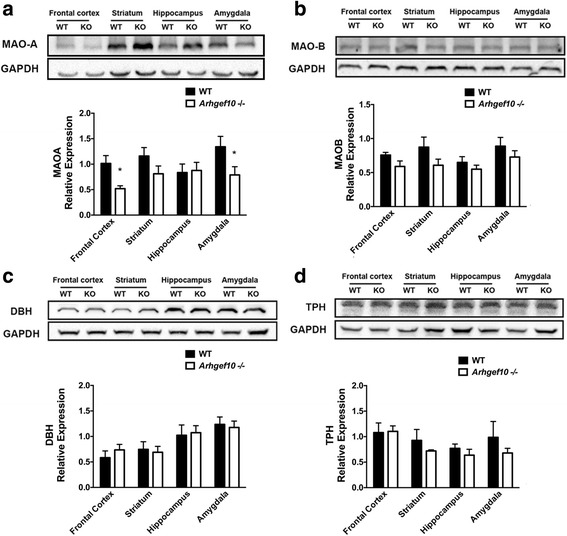
MAO-A is decreased in *Arhgef10* ^−^/^−^ mice. The main degradation enzymes 5-HT and NE were analyzed by Western blotting. **a** MAO-A expression was decreased in the frontal cortex and amygdala of *Arhgef10* ^−^/^−^ mice. Another metabolizing enzyme, MOA-B, did not show a significant difference between WT and KO mice **b**. The main enzymes for the synthesis of the amine neurotransmitters were also analyzed by Western blotting. The protein levels of both DBH **c** and TPH **d** were similar between WT and KO mice in the examined brain areas. *p < 0.05 compared with WT. *MAO-A* monoamine oxidase A, *MAO-B* monoamine oxidase B, *DBH* dopamine β-hydroxylase, *TPH* tryptophan hydroxylase

Since NE and 5-HT levels were significantly elevated in *Arhgef10* knockout mice, we measured the expression of MAO-A and MAO-B, the key enzymes that degrade NE and 5-HT, in the corresponding brain areas using Western blotting. MAO-A levels between WT and *Arhgef10* knockout mice were evaluated by two-way ANOVA (two-way ANOVA for genotype × brain regions, main effect of genotype: *F*_1, 12_ = 4.305, *p* = 0.0602 and brain regions *F*_3, 36_ = 2.286, *p* = 0.0953; interaction: *F*_3, 36_ = 2.324, *p* = 0.0913). Post hoc comparisons between WT and KO revealed significant differences in frontal cortex and amygdala. Further comparison of these differences found significantly reduced MAO-A levels in the frontal cortex and amygdala (Fig. 7a). In contrast with MAO-A levels, no statistically significant difference was found in MAO-B levels between WT and *Arhgef10* knockout mice (two-way ANOVA for genotype × brain regions, main effect of genotype: *F*_1, 4_ = 3.163, *P* = 0.1499 and brain regions *F*_3, 12_ = 2.688, *p* = 0.0934; interaction: *F*_3, 12_ = 0.3990, *p* = 0.7563) (Fig. 7b). Furthermore, we also examined the enzymes dopamine β-hydroxylase (DBH) and tryptophan hydroxylase (TPH), which are involved in the synthesis of NE and 5-HT, respectively. WT and *Arhgef10* knockout mice had similar protein levels of both DBH (two-way ANOVA for genotype × brain regions, main effect of genotype: *F*_1, 4_ = 0.03559, *p* = 0.8595 and brain regions *F*_3, 12_ = 7.614, *p* = 0.0041; interaction: *F*_3, 12_ = 0.2854, *p* = 0.8350) and TPH (two-way ANOVA for genotype × brain regions, main effect of genotype: *F*_1, 4_ = 0.5692, *p* = 0.4926 and brain regions *F*_3, 12_ = 6.591, *p* = 0.0070; interaction: *F*_3, 12_ = 1.190, *P* = 0.3549) in the tested brain regions (Fig. 7c, d).

## Discussion

In a previous clinical study, we found a terminal deletion containing the gene *ARHGEF10* on chromosome 8 in autistic patients. The current study demonstrates that loss of function of *Arhgef10* leads to impairment of social behavior and altered mood-related behavior in mice. Importantly, *Arhgef10* knockout mice showed indifference toward the novel objects and stranger mice in the free social interaction assay, as well as a lack of preference between the novel mouse and the familiar mouse during the social recognition test. Overall, *Arhgef10* knockout mice displayed no preference between the empty chamber and stranger mouse 1 and showed no preference for social interaction with the novel mouse, stranger 2. These results indicate that ARHGEF10 deficiency might cause a deficit in social interaction and social recognition. Interestingly, although *Arhgef10* knockout mice stayed longer in the chamber with the first stranger mouse than the chamber with the second stranger mouse (Fig. 2d), there was no difference in the time of social interaction between the two chambers containing stranger mice (Fig. 2e). These results suggest that *Arhgef10* knockout mice lacked interest in interaction with stranger mice. Moreover, *Arhgef10* knockout mice exhibited a decreased tendency to explore the area with the second mouse. This decreased tendency to investigate the area with a novel stranger mouse may correlate with an aloof character typical of autism. The reduced social interaction and lack of preference for social novelty observed in the three-chamber social behavior tests support our speculation that the deletion of ARHGEF10 might partly be correlated with clinical manifestations in individuals with ASD.

Several published observations have shown that children with ASD are at a high risk of anxiety disorders [21]. In this study, a reduced anxiety level was found in knockout mice, compared with WT mice, in the EPM test. *Arhgef10* knockout mice spent a longer duration in the open arms than WT mice, implying reduced anxiety-like behavior in *Arhgef10* knockout mice. Moreover, the reduced anxiety-like behavior in EPM could result from the decreased innate fear responses to elevated and open areas. Additionally, *Arhgef10* knockout mice entered the open arm more frequently than WT, which suggests *Arhgef10* deficiency did not affect the natural tendency to explore novel places. However, in the three-chamber test, the tendency to investigate the place with a novel stranger was reduced in *Arhgef10* knockout mice, indicating possible social withdrawal behavior. Although the finding of a lower level of anxiety in *Arhgef10* knockout mice is inconsistent with another reported autism-like mouse strains [22, 23], the reduced anxiety in *Arhgef10* knockout mice indicates that social interaction defects were not caused by anxiety-like behavior. The reduction of anxiety-like behavior strengthened the correlation between *Arhgef10* and social behavior and suggests the involvement of *Arhgef10* in anxiety-related behavior.

We further evaluated specific depression-like behavior using the FST and the TST, to verify that social deficits in *Arhgef10* knockout mice are not caused by depression-related effects. Compared with WT mice, *Arhgef10* knockout mice exhibited lower immobility time in the FST and the TST than WT mice, suggesting that *Arhgef10* KO reduces depression-like behavior. These results revealed that ARHGEF10 is also involved in the regulation of depression-like behavior. The lower levels of anxiety-like and depression-like behavior further demonstrated that the social traits in *Arhgef10* knockout mice were not the results of mood-related behavioral abnormalities. In addition, *Arhgef10* knockout mice exhibited enhanced locomotor activity in a novel open field, which might be a reflection of the hyperactivity that is often observed clinically in autistic patients. The features of hyperactivity have also been reported in other animal models of autism [24–26]. Although *Arhgef10* knockout mice displayed characteristic hyperactivity, the number of entries into each compartment was not increased in the three-chamber tests, indicating that the reduced social behavior was not a result of hyperactivity. When the results of social impairment and hyperactivity are taken together, the ASD-associated social inability observed in the *Arhgef10* deficiency mice appears not to result from low levels of spontaneous activity. The reduced anxiety-like behavior and hyperactivity phenotype observed in *Arhgef10* knockout mice may be a sign of increased impulsive behavior. We thus further evaluated impulsive behavior using the electro-foot shock aversive water drinking test (EFSDT) [27]. *Arhgef10* knockout mice displayed no obvious impulsive behaviors in comparison with WT mice (Additional file 2: Figure S2-2).

Although some studies suggest that autism features sensorimotor deficits [28], we did not find any PPI deficit in *Arhgef10* knockout mice. No abnormalities were found in the startle response or sensory gating, indicating that the *Arhgef10* knockout does not affect the sensory gating system in mice. Moreover, escape latencies were comparable between WT and *Arhgef10* knockout mice in the Morris water maze, suggesting that the deletion of ARHGEF10 does not impair spatial-learning-based behavior.

Social behavior can be affected by many behavioral factors, including anxiety levels, spontaneous activity, depression, sensory perception, memory, and cognition. *Arhgef10* knockout mice displayed normal sensorimotor responses in the PPI test and normal spatial learning behavior in the water maze. Moreover, *Arhgef10* knockout mice did not exhibit depression-like or anxiety-like behavior, suggesting that the social deficit was not confounded by these factors. Our study demonstrated that the loss of function of ARHGEF10 mainly caused social impairment in mice.

In this study, changes in neurotransmitter levels were observed as well. Serotonin and norepinephrine levels were significantly increased in the frontal cortex and amygdala of *Arhgef10* knockout mice. The amygdala has been implicated in various functions concerning emotion and social behavior. Several studies in primates and other mammalian species have implicated the amygdala as an important contributor to social behavior [29, 30]. Neonatal lesions of the amygdala in rats results in a social behavior deficit, and these rats exhibit autistic-like symptoms [31]. Furthermore, an early bilateral lesion of the amygdala in rhesus monkeys leads to reduced social interactions with other monkeys [32]. On the basis of the biological function of the amygdala, it is proposed to be one of the main regulatory brain regions driving the pathology of ASD [33]. Furthermore, the frontal cortex is the region responsible for emotion control and decision making and has been reported to be associated with the cognitive and anatomical abnormalities in patients with autism [34]. Based on the distinctive roles of the frontal cortex and amygdala in autism, the neurochemical changes in these brain regions may be linked to the observed phenomena in *Arhgef10* knockout mice. The neurochemical study of autism began with a report of changes in serotonin [35]. An elevated serotonin level in whole blood is a well-characterized biomarker in autism research [6]. Additionally, increased serotonin synthesis capacity has been reported in autistic children, suggesting that serotonin may play an important role in the development of autism [36]. The elevated serotonin levels found in the brains of *Arhgef10* knockout mice correlate with attenuated depression-like behaviors observed in the FST and the TST. These findings also support the role of the serotonin system in some autism patient symptoms. Apart from serotonin, norepinephrine is also an important monoamine associated with neuronal functions and levels of anxiety, arousal, and stress sensitivity, and many of these functions are also impaired in ASD. Evidence for involvement of norepinephrine in ASD comes from a report indicating that patients with autism and their families showed elevated plasma levels of NE and low enzyme activity [37]. The Angelman syndrome mouse model, which exhibits autistic-like behavior, shows increased norepinephrine and serotonin levels in different brain regions [23]. In addition, the elevated serotonin level in brain found in *Arhgef10* knockout mice can also explain their attenuated depression-like behaviors in the FST and the TST. The changes of serotonin and norepinephrine in the frontal cortex and amygdala indicate that ARHGEF10 might play a role in linking the phenotype of social impairment to the balance of monoamines. Therefore, the loss of homeostasis of serotonin and norepinephrine in the frontal cortex and amygdala of the *Arhgef10* knockout mice might be the basis of their autistic-like behaviors.

We sought to further understand the underlying mechanisms that contribute to the increased level of amine neurotransmitters. Therefore, we examined MAO-A and MOA-B, which are the key enzymes responsible for catalyzing the metabolism of monoamines, including serotonin and norepinephrine. In addition, the enzymes dopamine β-hydroxylase (DBH), and tryptophan hydroxylase (TPH), which are responsible for the synthesis of norepinephrine and serotonin, respectively, were also examined using Western blot. MAO-A was decreased in the frontal cortex and amygdala in *Arhgef10* knockout mice. However, the levels of DBH and TPH were comparable to those of WT littermates, indicating that the synthesis of the corresponding neurotransmitters was not affected by the *Arhgef10* knockout. The lower level of MAO-A may reduce the capacity for serotonin and NE degradation. The decreased MAO-A in *Arhgef10* knockout mice provides a possible explanation for the higher levels of serotonin and norepinephrine in the frontal cortex and amygdala. Recently, a study demonstrated that patients with autism had decreased MAO-A activity in the frontal cortex and cerebellum [38]. Moreover, mice lacking MAO-A displayed increased serotonin and norepinephrine levels and aggressive behavior [39]. Another knockout mouse lacking MAO-A/B also exhibited elevated serotonin levels and autistic-like features [40]. These previous reports support our findings in *Arhgef10* knockout mice, which implied that the reduced MAO-A levels and elevated amine levels might be the causes of social impairment. Although the phenotypes might result from changes in amine levels in the frontal cortex and amygdala, further study of the neurotransmitters and related molecules that are regulated by ARHGEF10 is required to clarify the underlying mechanism.

## Conclusion

In conclusion, this study demonstrated the behavioral and neurochemical changes of *Arhgef10* knockout mice. These mice exhibited social inability in the three-chamber test, reflecting the social impairment observed in humans with ASD. On the other hand, *ARHGEF10* knockout mice displayed hyperactivity in the locomotor test, reduced anxiety-like behavior in the EPM, and reduced depression-like behavior in the FST and TST. These behavioral changes further confirmed that the social deficits in *Arhgef10* knockout mice were not confounded by mood disorders. Moreover, neurochemical changes such as elevated serotonin and norepinephrine levels were also found in the frontal cortex and amygdala in *Arhgef10* knockout mice. Further analysis of MAO-A and MAO-B, the key enzymes that degrade norepinephrine and serotonin, revealed that the loss of function of ARHGEF10 decreased the expression of MAO-A in the frontal cortex and amygdala. These results suggest that *ARHGEF10* is a risk gene for ASD, especially correlated with the symptom of social activity impairment.

## Additional file

Additional file 1: Generation of ARHGEF10 -/- mice. (PPTX 46 kb)

Additional file 2: Impulsive and Non-impulsive behavior. (PDF 15155 kb)

## Abbreviations

5-HT: Serotonin
ARHGEF10: A rho guanine nucleotide exchange factor 10
ASD: Autism spectrum disorder
CNV: Copy number variation
DBH: Dopamine ß-hydroxylase
EFSDT: Electro-foot shock aversive water drinking test
EPM: Elevated plus maze
FST: Forced swimming test
GAPDH: Glyceraldehyde-3-phosphate dehydrogenase
GEF: Guanine nucleotide exchange factor
KO: Knockout
MAO-A: Monoamine oxidase A
MAO-B: Monoamine oxidase B
NE: Norepinephrine
PPI: Pre-pulse inhibition
TPH: Tryptophan hydroxylase
TST: Tail suspension test
WT: Wild-type
