# Supplementary material for: Impairment of social behaviors in Arhgef10 knockout mice
Source: Mol Autism. 2018 Feb 13;9:11. doi: 10.1186/s13229-018-0197-5 (PMC5810065; doi:10.1186/s13229-018-0197-5)
Supplement: Supplementary file 1 — Generation of ARHGEF10 -/- mice. (PPTX 46 kb) [file 13229_2018_197_MOESM1_ESM.pptx]

## Slide 1
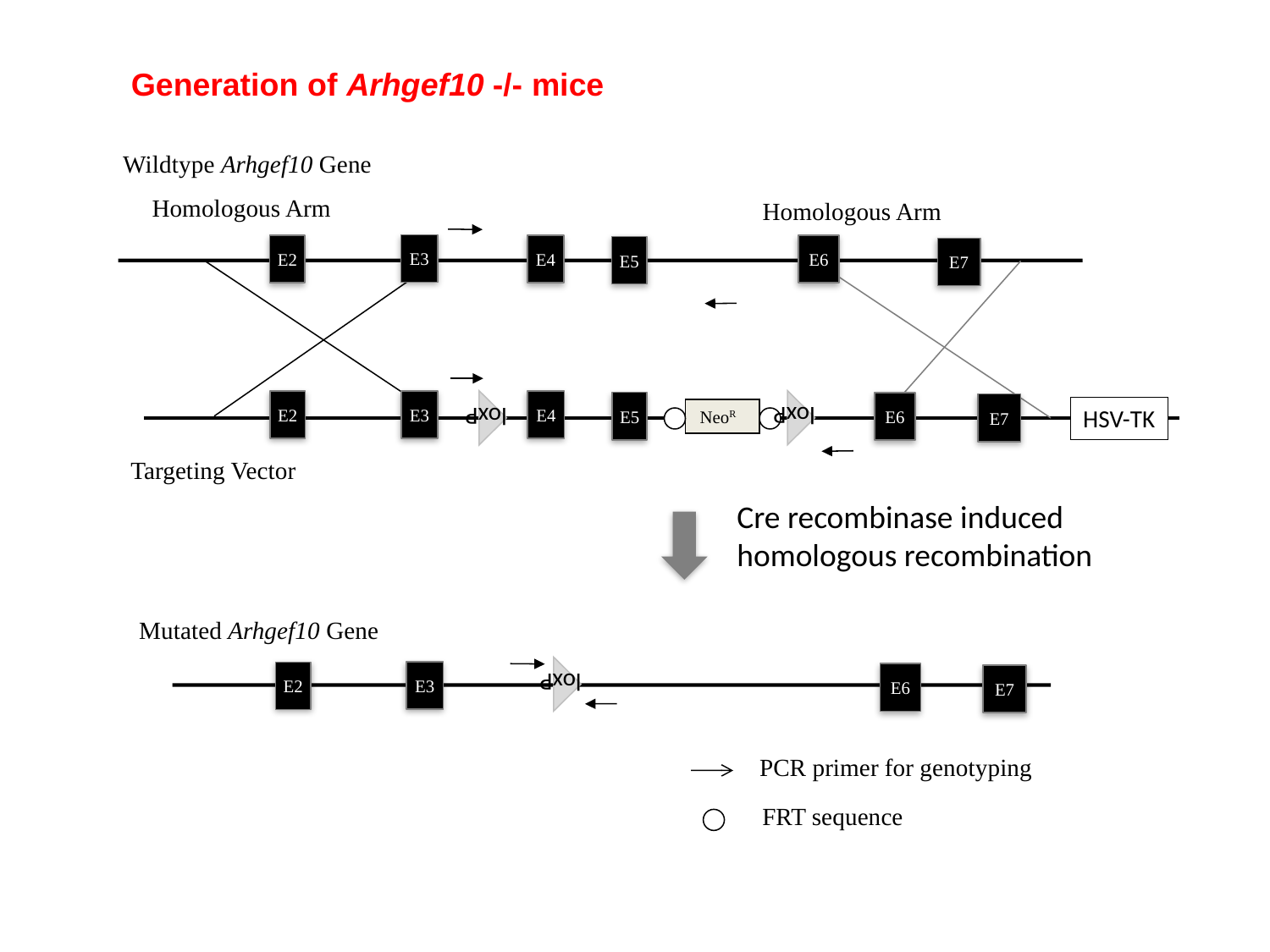

Generation of Arhgef10 -/- mice
Wildtype Arhgef10 Gene
Homologous Arm
Homologous Arm
E3
E2
E4
E6
E5
E7
E3
E2
E4
E5
E6
E7
HSV-TK
NeoR
loxP
loxP
Targeting Vector
Cre recombinase induced homologous recombination
Mutated Arhgef10 Gene
E3
E2
E6
E7
loxP
 PCR primer for genotyping
FRT sequence
