# Supplementary material for: Impairment of social behaviors in Arhgef10 knockout mice
Source: Mol Autism. 2018 Feb 13;9:11. doi: 10.1186/s13229-018-0197-5 (PMC5810065; doi:10.1186/s13229-018-0197-5)
Supplement: Supplementary file 2 — Impulsive and Non-impulsive behavior. (PDF 15155 kb) [file 13229_2018_197_MOESM2_ESM.pdf]

S2. Material and Method

In order to examine the impulsive behavior in *Arhgef10* knockout mice, the elcetro-foot shock aversive water drinking test (EFSDT) was used as described by Kim et al. (2012) with some modifications. In brief, a black Plexiglas box sized 40 x 40 x 30 cm was divided into three compartments (Start area, water area and free area). The floor of the water area was made of electrified grid wire and a water bottle was fitted from outside of the black box at 4 cm height in the water area. There were two sessions in the EFSDT, including training session and test session. Mice were deprived of water at least 18 hours in the home cage during the EFSDT. In the training session (Day-1 and Day-2), the mice were trained to licked water from the bottle in the water area. When the mice licked water more than 5 seconds, they were removed from the water area by the experimenter. Each training session consisted of 2 trials for 10 minutes (separated at least 60 minutes) for two consecutive days. During the test session (Day-3), an electric-shock (0.2 mA, 0.5 second, from stimulator, Model 2100, A-M Systems, Carlsborg, WA, USD) was given when the mice licked water more than 5 seconds. The drinking behavior resulting in electroshock was defined as impulsive drinking. On the other hand, the mice drinking within 5 seconds was defined as non-impulsive drinking. The numbers of impulsive drinking and non-impulsive drinking were counted within 10 min.

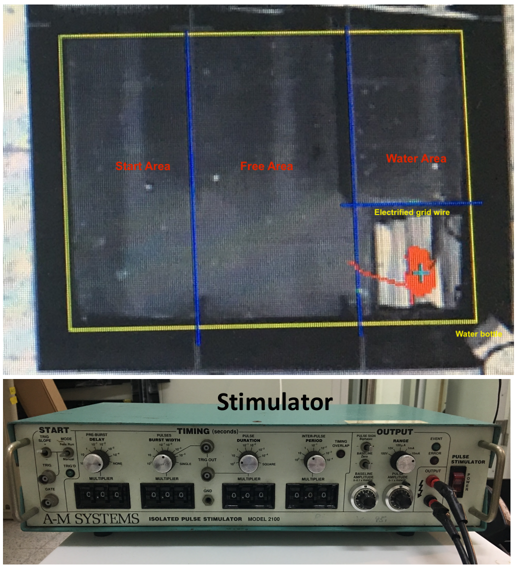

S2-1 Impulsive behavior testing apparatus

Results

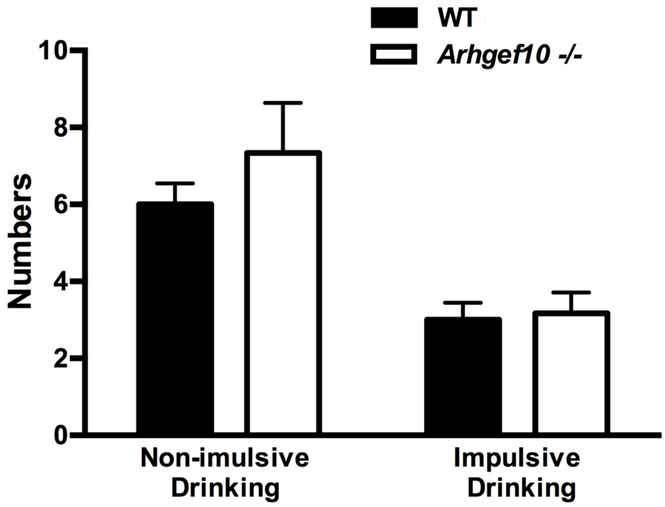

S2-2. No impulsive behavior in *Arhgef10* knockout mice

Arhgef10 knockout mice had a comparable numbers of non-impulsive and impulsive drinking as WT mice. (n=4 and 6 for WT and *Arhgef10* -/- mice, respectively)
